# When motivation is not enough: the moderating role of social support in cross-cultural adaptability among Chinese students learning Arabic

**DOI:** 10.3389/fpsyg.2026.1779106

**Published:** 2026-02-11

**Authors:** Yanting Huang, Jiwen Chen

**Affiliations:** 1 School of Eastern Languages, Hainan College of Foreign Studies, Wenchang, Hainan, China; 2 Dongshin University, Naju-si, Republic of Korea

**Keywords:** Arabic language learners, China, cross-cultural adaptability, cultural intelligence, learning motivation, moderating effect, social support

## Abstract

**Introduction:**

Against the backdrop of the Belt and Road Initiative, the demand for professionals proficient in Arabic and capable of cross-cultural communication has surged, yet the mechanism of how learners navigate the significant cultural distance remains underexplored. This study aims to investigate the relationship between learning motivation and cross-cultural adaptability among Chinese Arabic language learners, specifically examining the moderating role of social support.

**Methods:**

Adopting a quantitative survey design, data were collected from 315 undergraduate Arabic majors. The study employed Confirmatory Factor Analysis (CFA), hierarchical multiple regression, and Instrumental Variable Two-Stage Least Squares (IV-2SLS) analysis to test the hypotheses.

**Results:**

Findings indicate that both learning motivation (B = 0.319, *p* < 0.001) and social support (B = 0.494, *p* < 0.001) significantly and positively predict cross-cultural adaptability. Crucially, social support functions as a significant moderator (B_interaction = 0.703, *p* < 0.001), with the final model explaining 74.2% of the variance. Simple slope analysis reveals that the positive association between motivation and adaptability is significant only under high levels of social support, acting as a necessary boundary condition. Further analysis based on Self-Determination Theory subtypes specifically highlights that this moderating effect is most pronounced for external regulation, suggesting that learners driven by instrumental goals are particularly dependent on external resources to translate motivation into adaptive outcomes. Robustness checks using IV-2SLS (B = 0.630) confirmed the stability of these associations.

**Discussion:**

Theoretically, this study extends the existing literature by identifying the boundary conditions under which motivation predicts adaptability, suggesting that internal drive and external support function together to facilitate adaptation. Practically, the findings indicate that higher education institutions should provide multi-dimensional support systems to assist students in translating their motivation into effective cross-cultural competence.

## 1 Introduction

With the deepening of China's Belt and Road Initiative, the strategic partnership between China and Arab countries has entered a new historical phase, characterized by extensive cooperation in areas such as politics, economics, and culture (Huwaidin, 2022). Against this backdrop, the demand for professionals proficient in Arabic and capable of high-level cross-cultural communication has surged. These professionals serve not only as language transmitters but also as bridges connecting Chinese and Arab civilizations (Al-Nahdi and Zhao, 2022). However, Arabic, with significant differences from Chinese in terms of language family, writing system, thinking patterns, and cultural background, presents unique challenges for Chinese students (Dajani et al., 2014). Beyond the complexity of the language itself, learners must also face and adapt to the divergent Arab culture, which can lead to culture shock and adaptation difficulties, thereby affecting learning outcomes (Darvin and Norton, 2021; Keser et al., 2023).

In this context, two core constructs have emerged: Learning Motivation and Cross-Cultural Adaptability. Learning motivation, defined not merely as a driving force but as a complex self-regulatory mechanism governing the direction and persistence of learning behavior, is widely regarded as a key predictor of success in second language acquisition (Dörnyei and Ushioda, 2021; Kai Liao et al., 2021). However, previous research has mainly focused on the effects of motivation on cognitive aspects such as language proficiency and language skills, with less attention given to its role in the more challenging social-psychological domain of cultural adaptation (Wei, 2023). Cross-cultural adaptability, which represents a multidimensional capacity to effectively manage psychological stress and behavioral appropriateness in unfamiliar cultural environments (Kelley and Meyers, 1995; King et al., 2021), is commonly discussed in terms of both psychological adaptation (e.g., emotional well-being and stress management) and sociocultural adaptation (e.g., behavioral competence and effective functioning in intercultural interactions), and the present study treats cross-cultural adaptability as encompassing these complementary facets (Taguchi, 2015). Therefore, a key scientific question is: Under what conditions does strong learning motivation effectively translate into robust cross-cultural adaptability?

Although it is intuitively plausible that learners with stronger motivation may actively engage in cross-cultural interactions, this process is not automatic. The learner's environment, particularly the social support they perceive, plays a crucial role (Wu et al., 2021). Social support, encompassing emotional, informational, and material support from family, friends, and mentors, is a well-recognized protective factor in helping individuals cope with stress and promoting psychological well-being (Zimet et al., 1988; Yan et al., 2025). However, in foreign language learning contexts, learning motivation and social support are still frequently examined as parallel predictors, and fewer studies have explicitly tested whether perceived social support moderates the motivation–adaptability association, particularly among domestic university students learning a linguistically and culturally distant language such as Arabic in China. For Arabic language learners facing cultural differences and learning pressures, the specific function of social support in the conversion of motivation into adaptability remains a nuanced theoretical gap. Drawing on the Stress-Buffering Model and Conservation of Resources theory, this study conceptualizes social support as a contextual resource that may reduce adaptation-related strain and facilitate the translation of motivational resources into sustained engagement and adaptive coping (Cohen and Wills, 1985). At the same time, consistent with Self-Determination Theory, learning motivation differs in quality, including intrinsic, identified, introjected, and external regulation; accordingly, learning motivation is operationalized as an overall composite index, and we further examine SDT-consistent motivational types to explore whether different forms of motivation show distinct patterns in their associations with cross-cultural adaptability.

Based on this analysis, this study explores three core questions: Does overall learning motivation (composite index) significantly positively predict cross-cultural adaptability (including psychological and sociocultural facets)? Does social support significantly positively predict cross-cultural adaptability? Does social support moderate the relationship between overall learning motivation and cross-cultural adaptability, and do supplementary SDT-based analyses indicate similar moderation patterns across different motivational qualities?

This study aims to construct and test a moderating model to investigate the impact of learning motivation on cross-cultural adaptability among Arabic language learners in Chinese universities. This population is theoretically and practically relevant, as Arabic entails substantial linguistic and cultural distance, requiring engagement with unfamiliar cultural norms even in domestic contexts. Under the Belt and Road Initiative, Arabic majors are expected to serve as future mediators in China–Arab exchanges, rendering cross-cultural adaptability an essential outcome beyond language proficiency. Importantly, we introduce social support as a key moderating variable to reveal the role of external environmental resources in the process of psychological and behavioral transformation.

Through this research, we aim to contribute in two key areas: Previous studies have confirmed the independent effects of learning motivation (as an intrinsic driver) and social support (as an external resource) on language learning and adaptation (Dörnyei and Ushioda, 2021; Chen, 2025). However, these studies largely treat the two factors as parallel influences, leaving the interaction mechanism between individual intrinsic motivation and external support environments less defined. This study extends the literature by integrating Self-Determination Theory (SDT) and the Stress-Buffering Model of social support. Our proposed moderating model shifts the focus from examining the “main effects” of variables to exploring their “interaction effects.” This approach helps provide a more integrated explanatory mechanism and offers an incremental refinement to sociocultural adaptation research by specifying when learning motivation is more likely to translate into cross-cultural adaptability under different levels of perceived support.

This study provides insights into how learning motivation and social support interact to enhance cross-cultural adaptability. The findings highlight that social support strengthens the relationship between motivation and adaptability, especially for students with external regulation. The results suggest that interventions should be tailored to students' motivational profiles. For students with high external regulation and low support, academic guidance and recognition are crucial, while for those with high intrinsic motivation but low support, emotional support and peer engagement are key. These findings can help universities design more targeted curricula and support systems, fostering both language proficiency and cultural competence in alignment with China's Belt and Road Initiative.

Section 2 reviews the relevant literature and develops the research hypotheses based on Self-Determination Theory, cross-cultural adaptation theory, and the stress-buffering model of social support. Section 3 describes the research design, participants, instruments, and data analysis procedures. Section 4 presents the empirical results, including the main effects, moderation analyses, and robustness checks. Section 5 discusses the findings in relation to existing literature and outlines theoretical and practical implications, acknowledging limitations, and suggesting directions for future research. Finally, Section 6 summarizes the conclusions.

## 2 Literature review and hypotheses development

### 2.1 Integration of self-determination theory, cultural adaptation, and social support

The theoretical framework for this study is built on three pillars: Self-Determination Theory (SDT), Sociocultural Adaptation Theory, and the Stress-Buffering Hypothesis of social support.

Self-Determination Theory (SDT) provides a foundation for understanding learning motivation. proposed by Deci and Ryan (2000) suggests that motivation exists on a continuum from external control to full autonomy. The theory posits that satisfying three basic psychological needs—autonomy, competence, and relatedness promotes intrinsic motivation and self-determined extrinsic motivation. In this study, learning motivation is measured using a composite index, with the further analysis differentiating four types of motivation based on their quality and relevance to cross-cultural adaptability: Intrinsic motivation, driven by interest and enjoyment of the activity itself; Identified regulation, driven by personal values and the recognition of the activity's importance; Introjected regulation, driven by internal pressures such as guilt or the desire for approval; External regulation, driven by external rewards or avoiding punishment (Chen et al., 2021).

Sociocultural Adaptation Theory focuses on individuals‘ ability to adapt to new cultural environments. According to Ward and Kennedy (1999), cross-cultural adaptation involves both psychological adaptation (emotional well-being) and sociocultural adaptation (behavioral competence in social interactions). This study conceptualizes cross-cultural adaptability as a comprehensive construct, incorporating both psychological and sociocultural dimensions, based on a widely recognized and authoritative cross-cultural adaptability scale. This scale allows for a holistic measurement of individuals' ability to manage emotional challenges, interact effectively in new cultural contexts, and adapt their behaviors in social interactions (Mepham and Martinovic, 2017).

The Stress-Buffering Hypothesis of Social Support explains how social support influences adaptation. This hypothesis posits that social support not only enhances well-being directly but also buffers the negative effects of stress (such as culture shock) by providing emotional comfort, informational advice, and practical help (Cohen and Wills, 1985). This study conceptualizes social support as a moderating factor, influencing how motivation translates into adaptability by reducing stress and offering resources that facilitate engagement and coping.

Integrating these three theories, we propose that learning motivation (from SDT) serves as the internal driver encouraging learners to engage in cross-cultural experiences and overcome challenges. Cross-cultural adaptability (from sociocultural adaptation theory) is the outcome that determines success in intercultural interactions. Social support (from the stress-buffering hypothesis) functions as an external resource that influences the extent to which motivation is translated into adaptability by providing support in times of stress and offering resources that facilitate sustained engagement and coping.

### 2.2 Learning motivation and cross-cultural adaptability

Learning motivation, especially intrinsic motivation driven by interest and value alignment, is widely regarded as the core predictor of success in language learning (Gardner, 2010). This study argues that the influence of motivation extends beyond language skill improvement and deeply permeates the social-psychological adaptation of learners. High-motivation learners tend to exhibit stronger perseverance, deeper learning strategies, and a more open and exploratory attitude toward the target culture (Dörnyei and Chan, 2013). This psychological orientation itself acts as a catalyst for developing cross-cultural adaptability (CCA).

The mechanism of this influence can be explained on both psychological and behavioral levels. Psychologically, strong learning motivation, especially highly internalized integrated motivation, can fundamentally reshape learners' cognitive framework for cross-cultural contexts (Xu et al., 2021). It encourages individuals to reframe potential cultural conflicts and uncertainties from “threats” to “challenges” and “learning opportunities,” thereby significantly enhancing their willingness to communicate in cross-cultural situations (MacIntyre and Legatto, 2010). This shift from an avoidance-oriented mindset to an approach-oriented one is the psychological foundation for initiating adaptive behaviors. On the behavioral level, this high psychological willingness systematically translates into higher frequency and quality of cross-cultural engagement. These behaviors are not isolated events but part of an ongoing, dynamic process where individuals actively and iteratively refine their cultural schemas (Aladini and Gheisari, 2025). We hypothesize that:

**H1**: Learning motivation significantly positively influences cross-cultural adaptability.

### 2.3 Social support and cross-cultural adaptability

Social support, referring to the care, help, and validation an individual perceives from their social network, is a key external resource for coping with stress and promoting psychological well-being (Cohen and Wills, 1985). In the field of cross-cultural adaptation, its importance has been repeatedly validated in numerous studies (Chen, 2025). Social support directly promotes adaptability by meeting learners' basic psychological needs. A supportive social environment (including affirmation from teachers and acceptance from peers) can effectively fulfill these needs, providing learners with a solid “psychological safety base” (Luan et al., 2020). On this base, learners' psychological resources for coping with loneliness and insecurity are released and redirected into more constructive and higher-order adaptive tasks.

Moreover, social support, as a crucial social capital, offers functional adaptation resources. Informational support, by providing normative knowledge and experiential strategies, reduces the ambiguity and uncertainty of new cultural environments, thereby accelerating cognitive adaptation and the acquisition of behavioral scripts (Rehman et al., 2020). Emotional support acts as a “stress buffer,” helping individuals maintain a positive self-concept and psychological resilience through empathy, encouragement, and value affirmation, effectively counteracting negative emotions induced by cultural differences and learning setbacks (Sawir et al., 2008). Thus, we hypothesize that:

**H2**: Social support significantly positively influences cross-cultural adaptability.

### 2.4 The moderating role of social support

We argue that social support not only directly promotes adaptability but also moderates (i.e., changes) the strength of the impact of learning motivation on cross-cultural adaptability. This moderating mechanism can be understood from the perspectives of Conservation of Resources Theory and the stress-buffering model. When individuals have abundant social support, they are better able to “invest” their psychological energy (e.g., motivation) into achieving higher-level goals, such as cross-cultural adaptability (Ng et al., 2017). A highly motivated learner without support may exhaust their psychological resources due to frequent frustration and isolation, preventing motivation from effectively translating into adaptability (DeFreese and Smith, 2013). Conversely, a highly motivated learner with a strong support network will be encouraged in their exploration, receive assistance in overcoming obstacles, and alleviate negative emotions. In such cases, social support acts as a “lever,” amplifying the initial strength of motivation (Hobfoll, 2001). We hypothesize that:

**H3**: Social support positively moderates the relationship between learning motivation and cross-cultural adaptability. Specifically, higher levels of social support enhance the positive influence of learning motivation on cross-cultural adaptability.

**Figure 1:**
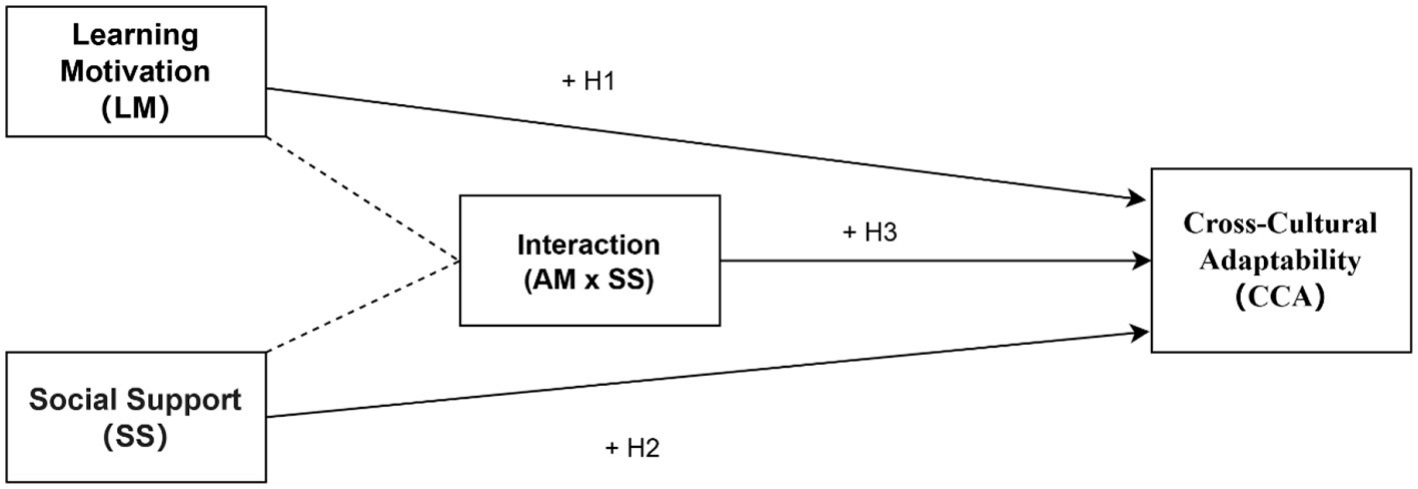
Theoretical framework: statistical moderation model.

Based on the above literature and theoretical assumptions, we have constructed a theoretical framework illustrating the direct relationships among learning motivation, social support, and cross-cultural adaptability. See Figure 1.

## 3 Research design and methodology

### 3.1 Participants and procedure

This study employed a combination of convenience sampling and snowball sampling to select participants from the Arabic language program at Yangzhou University in mainland China. While non-probability sampling inevitably limits the generalization of findings to the broader population (External Validity), focusing on this specific cohort in a high-intensity Arabic program ensures high ecological validity and allows for an in-depth examination of motivation-adaptation mechanisms within a clearly defined cultural context. The survey was distributed through the online questionnaire platform “Wenjuanxing,” with the assistance of Arabic language program instructors and student club leaders. Before the survey distribution, all participants were informed of the study's purpose, voluntary participation, anonymity, data usage, and signed an electronic informed consent form. Participants could withdraw from the survey at any time. To minimize social desirability bias and potential measurement errors inherent in self-report surveys, participants were assured that their responses would be confidential and used solely for academic research. A total of 342 questionnaires were collected, and after removing 27 invalid responses due to short completion times, pattern responses, or failure to pass screening questions, 315 valid questionnaires were retained, resulting in a valid response rate of 92.1%. The demographic characteristics of the sample are detailed in Table 1.

**Table 1:** Demographic characteristics of the sample (*N* = 315).

**Characteristic**	**Category**	**Frequency**	**Percentage, %**
Gender	Male	74	23.5
	Female	241	76.5
Age	18	78	24.8
	19	108	34.3
	20	111	35.2
	21	17	5.4
	22	1	0.3
Grade	Freshman	110	34.9
	Sophomore	108	34.3
	Junior	83	26.3
	Senior	14	4.4

Among the 315 participants, 74 were male (23.5%) and 241 were female (76.5%), reflecting the typical gender distribution in foreign language majors. The age range was from 18 to 22 years. In terms of grade distribution, 110 were freshmen (34.9%), 108 were sophomores (34.3%), 83 were juniors (26.3%), and 14 were seniors (4.4%).

### 3.2 Research instruments

All scales used in this study were translated and back-translated based on publicly available literature. To ensure clarity and applicability in the Chinese cultural context and specific learning situations, the scales were pretested with three Arabic language professors and five students. All scales employed a Likert 5-point rating scale (1 = Strongly Disagree, 5 = Strongly Agree).

#### 3.2.1 Learning motivation scale

This scale was developed based on Self-Determination Theory (SDT) and references Deci and Ryan (2000) and Noels et al. (2000). It includes 12 items. To address the multidimensional nature of motivation, this study adopted a dual analytical approach. First, consistent with previous L2 research, items were summed to form a composite measure of overall learning motivation intensity for the primary moderation model (Cronbach's α = 0.951). Second, to capture the qualitative differences in motivation emphasized by SDT, four subscales (Intrinsic, Identified, Introjected, and External Regulation) were calculated for further analysis.

#### 3.2.2 Cross-cultural adaptability inventory

The Chinese revised version of the CCAI developed by Kelley and Meyers (1995) was used. This scale is an authoritative tool for measuring cross-cultural adaptability and includes four dimensions: Emotional Resilience, Flexibility/Openness, Perceptual Acuity, and Personal Autonomy. Consistent with the conceptualization of adaptability as a holistic competence, the total score was used as the primary outcome variable. The overall Cronbach's α for this scale in this study was 0.964.

#### 3.2.3 Multidimensional scale of perceived social support

The Chinese version of the MSPSS developed by Zimet et al. (1988) was used. This scale is widely used to measure perceived social support from three sources: family, friends, and other significant others (defined in this study as teachers and mentors). The scale contains 12 items, such as “When I face difficulties, my family is really willing to help me.” The overall Cronbach's α for this scale in this study was 0.962.

#### 3.2.4 Control variables

Given that gender and grade level might influence foreign language learning and cultural adaptation, these three variables were included as control variables in the regression models.

### 3.3 Data analysis strategy

Data processing was conducted using SPSS and R software. The specific steps were as follows:

Descriptive Statistics: Descriptive statistics were performed on the demographic characteristics of the sample.

Control for Common Method Bias (CMB): Given the reliance on self-report measures, both procedural remedies (e.g., strict anonymity, clear instructions) and statistical checks (Harman's single-factor test and CFA model comparison) were employed to ensure data validity.

Confirmatory Factor Analysis (CFA): CFA was conducted using R software to test the measurement model fit for the three core constructs learning motivation, social support, and cross-cultural adaptability and to evaluate convergent and discriminant validity.

Descriptive Statistics and Pearson Correlation Analysis: Descriptive statistics and Pearson correlation analysis were conducted to explore the mean, dispersion, and relationships among the variables.

Hierarchical Multiple Regression: Hierarchical multiple regression was performed using SPSS to test the main associations and moderation hypotheses. Before constructing interaction terms, both the independent and moderator variables were centered.

Simple Slopes Analysis: If significant interaction effects were found, simple slopes analysis and moderation effect plots were generated to more intuitively reveal the moderation pattern.

Causal Inference Robustness Tests: To further support the hypothesized causal direction, two supplementary analyses were conducted:

a) Instrumental Variable Two-Stage Least Squares (IV-2SLS): To rigorously control for potential endogeneity of learning motivation (e.g., motivation may be related to unobservable personal traits like cultural sensitivity, or there may be a risk of bidirectional causality), the IV-2SLS method was used. “Whether Arabic was the first choice during the university entrance exam (Gaokao)” was selected as the instrument for learning motivation.

An effective instrument must satisfy two core assumptions: relevance and exclusion restriction. First, according to Self-Determination Theory (Deci and Ryan, 2000), autonomous choice is a key antecedent to intrinsic motivation; previous studies have shown that students who are admitted based on their first-choice major generally exhibit stronger commitment and enthusiasm toward their studies, ensuring a strong correlation between the instrument and the endogenous explanatory variable (learning motivation) (Kirkeboen et al., 2016). Second, filling out the Gaokao application is a pre-admission decision, and according to the identification strategy in educational economics, it is unlikely to directly influence the students' current cross-cultural adaptability through any other pathway except through learning motivation (i.e., their investment in the major), thus meeting the exclusion restriction (Barrow and Malamud, 2015).

b) Subgroup Robustness Checks: The sample was divided into two key demographic variables—grade level (low grade group vs. high grade group) and gender (male group vs. female group). The core hierarchical regression analysis was repeated within these subgroups to test whether the main and moderating effects hypotheses hold across different groups.

## 4 Data analysis and results

### 4.1 Common method bias test

Acknowledging the potential limitations of self-report measures, we employed both procedural and statistical remedies to control for Common Method Bias (CMB).

First, regarding procedural control, strict anonymity was assured during data collection to reduce evaluation apprehension, and we emphasized that there were no “right” or “wrong” answers to minimize social desirability bias.

Second, statistically, we conducted two tests: (1) Table 2 Harman's single-factor test indicated that the first principal component accounted for 38.474% of the variance, which is well below the 40% threshold. (2) A confirmatory factor analysis (CFA) comparison was performed to rigorously test for method effects (Podsakoff et al., 2003). As shown in Table 3, the One-Factor Model (loading all items onto a single construct) exhibited extremely poor fit (χ^2^ = 5511.03, df = 902, χ^2^/df = 6.11, CFI = 0.563, TLI = 0.542 RMSEA = 0.128). In sharp contrast, the hypothesized Three-Factor Model demonstrated excellent fit (χ^2^ = 963.05, df = 899, χ^2^/df = 1.07, CFI = 0.994, TLI = 0.994 RMSEA = 0.015). The significant deterioration in model fit when combining factors suggests that CMB is not a pervasive issue in this study and that the constructs are empirically distinct.

**Table 2:** Harman's single-factor test results.

**Factor**	**Initial eigenvalues**	**(%) (variance explained)**	**(%) (cumulative variance)**
1	16.929	38.474	38.474
2	8.683	19.734	58.208
3	2.656	6.036	64.244
…	…	…	…

**Table 3:** Comparison of measurement models for test of common method bias.

**Model**	**χ^2^**	**df**	**χ^2^/df**	**CFI**	**TLI**	**RMSEA**
One-factor model	5,511.03	902	6.11	0.563	0.542	0.128
Two-factor model	4,369.35	901	4.85	0.671	0.655	0.111
Three-factor model	963.05	899	1.07	0.994	0.994	0.015

### 4.2 Confirmatory factor analysis

Additionally, Table 4 reports the reliability and convergent validity indicators. The standardized factor loadings for all constructs ranged from 0.606 to 0.778, and all were statistically significant (*p* < 0.001). Cronbach's α coefficients and Composite Reliability (CR) values exceeded the recommended threshold of 0.800, suggesting high internal consistency. The Average Variance Extracted (AVE) for each construct exceeded 0.500, indicating satisfactory convergent validity.

**Table 4:** Confirmatory factor analysis: reliability and convergent validity.

**Construct and item**	**Std. loading**	**Cronbach's α**	**CR**	**AVE**
LM		0.951	0.951	0.617
LM1	0.768			
LM2	0.777			
… (Other item)	…			
AM12	0.775			
SS		0.962	0.962	0.678
SS1	0.817			
SS2	0.830			
… (Other item)	…			
SS12	0.839			
CCA		0.964	0.965	0.578
CCA1	0.785			
CCA2	0.789			
… (Other item)	…			
CCA20	0.762			

### 4.3 Descriptive statistics and correlation analysis

Table 5 presents the means, standard deviations, and correlation matrix for the main variables. The results show that the mean score for learning motivation was 3.911 (SD = 0.557), cross-cultural adaptability was 3.509 (SD = 0.500), and social support was 3.650 (SD = 0.629). These scores indicate that participants generally reported levels above the midpoint on these variables.

**Table 5:** Means, standard deviations, and correlation matrix for key variables (*N* = 315).

**Variable**	**M**	**SD**	**1**	**2**	**3**	**4**	**5**
1. Gender^a^	1.77	0.425	1				
2. Grade	2	0.890	0.002	1			
3. LM	3.911	0.557	−0.070	−0.023	1		
4. SS	3.650	0.629	−0.060	−0.048	−0.094	1	
5. CCA	3.509	0.500	−0.062	0.005	0.358^**^	0.581^**^	1

^a^Gender: 1 = male, 2 = female.

LM, Learning Motivation; SS, Social Support; CCA, Cross-Cultural Adaptability.

^**^p < 0.01.

The correlation analysis results indicate that: Learning motivation was significantly and positively associated with cross-cultural adaptability (r = 0.358, *p* < 0.01). Social support was significantly and positively associated with cross-cultural adaptability (r = 0.53, *p* < 0.001). Learning motivation was significantly and positively associated with social support (r = 0.581, *p* < 0.01).

These results provide preliminary support for the hypothesized relationships tested in the subsequent analysis.

### 4.4 Hypothesis testing: hierarchical regression analysis

To examine the relationships between learning motivation (intrinsic factor), social support (extrinsic factor), and cross-cultural adaptability, as well as the potential moderating role of social support, a four-step hierarchical regression analysis was conducted. Demographic variables were controlled to minimize potential confounding effects. The detailed statistical results are presented in Table 6.

**Table 6:** Hierarchical regression analysis results for the moderating role of social support.

**Variable**	**Model 1**	**Model 2**	**Model 3**	**Model 4**
**Step 1: controlling variables**	**B(SE)**	**B(SE)**	**B(SE)**	**B(SE)**
Gender	−0.073 (0.066)	−0.044 (0.062)	0.05 (0.047)	0.006 (0.034)
Grade	0.003 (0.032)	0.007 (0.030)	0.025 (0.022)	0.009 (0.016)
**Step 2 independent variable**				
LM		0.319 (0.048)^***^	0.375 (0.036)^***^	0.390 (0.026)^***^
**Step 3 adjustment variables**				
SS			0.494 (0.032)^***^	0.462 (0.023)^***^
**Step 4: interactive items**				
LM × SS				0.703 (0.042)^***^
**Model statistics**				
R^2^	0.004	0.130	0.511	0.742
F	0.613	15.467^***^	81.078^***^	177.665^***^

The dependent variable is cross-cultural adaptability. The values in the table are unstandardized regression coefficients (B).

^***^p < 0.001.

Step 1: Baseline Model for Control Variables (Model 1) In Model 1, gender and grade were included as control variables. The results showed that the overall model fit was low and not statistically significant (R^2^ = 0.004, F = 0.613, *p* > 0.05). The regression coefficients for gender (β = −0.073) and grade (β = 0.003) were not significant. This suggests that demographic characteristics alone did not account for a significant portion of the variance in cross-cultural adaptability among Arabic language learners in this sample.

Step 2: Main Effect of Learning Motivation (Model 2) In Model 2, the independent variable “learning motivation” was added. The results showed a substantial increase in the explanatory power of the model (ΔR^2^ = 0.126, *p* < 0.001), and the model became statistically significant (F = 15.467, *p* < 0.001). Specifically, learning motivation was positively related to cross-cultural adaptability (B = 0.319, SE = 0.048, *p* < 0.001). This indicates that higher levels of intrinsic motivation are associated with better adaptability to new cultural environments. This finding supports Hypothesis H1, suggesting that learning motivation is a key internal correlate of cross-cultural adaptability.

Step 3: Incremental Effect of Social Support (Model 3) In Model 3, “social support” was introduced. This led to a significant increase in model explanatory power, with R^2^ rising to 0.511 (ΔR^2^ = 0.381, *p* < 0.001). The regression results indicated that social support was a significant positive predictor of cross-cultural adaptability (B = 0.494, SE = 0.032, *p* < 0.001), supporting Hypothesis H2. Notably, after including social support, the standardized coefficient for learning motivation remained significant and slightly increased (from 0.319 to 0.375). This suggests that social support functions as an independent external resource that, alongside intrinsic motivation, contributes to the variance in cross-cultural adaptability.

Step 4: Moderating Effect of Interaction (Model 4) Model 4 included the interaction term “learning motivation × social support” (centered). The interaction term yielded a significant positive regression coefficient (B = 0.703, SE = 0.042, *p* < 0.001), supporting Hypothesis H3. With the inclusion of the interaction term, the total explanatory power of the model reached 74.2% (R^2^ = 0.742). This suggests that the strength of the association between learning motivation and adaptability is contingent upon the level of social support.

In conclusion, the hierarchical regression analysis supported the proposed main and interaction hypotheses. The model's explanatory power (R^2^) increased substantially with the sequential addition of variables (0.4% → 13.0% → 51.1% → 74.2%), underscoring the relevance of the integrated “motivation-support” framework.

### 4.5 Simple slopes analysis

**Figure 2:**
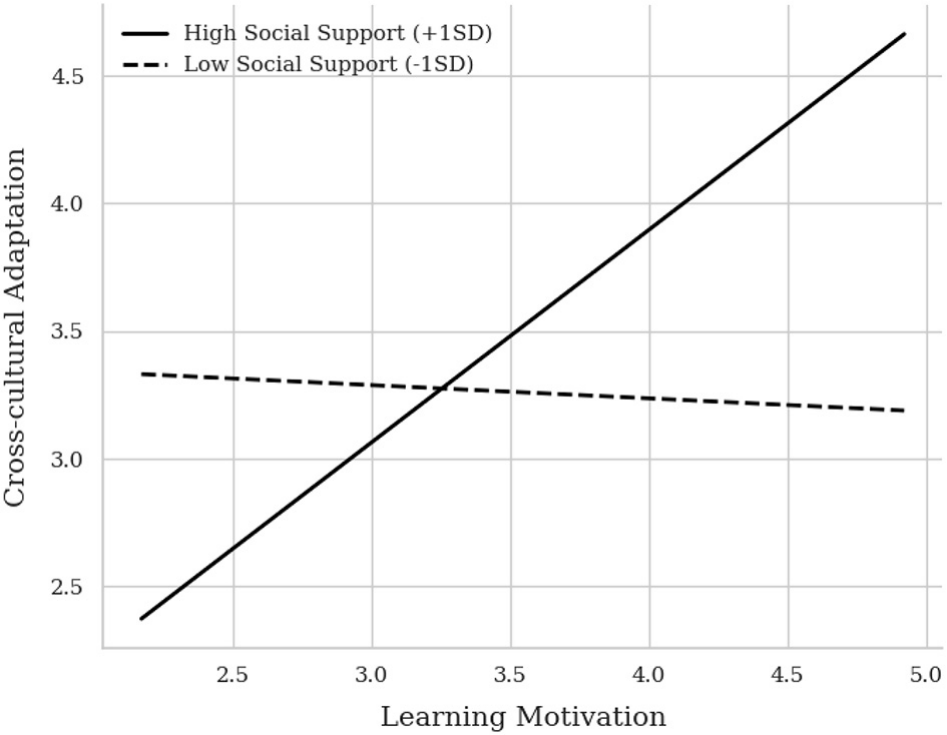
Simple slope plot of the moderating effect of social support.

To clarify the nature of the interaction effect, a simple slopes analysis was conducted (see Figure 2). The results indicated that at high levels of social support (mean + 1 SD), the positive association between learning motivation and cross-cultural adaptability was strong and statistically significant (B_simple = 0.833, *t* = 21.935, *p* < 0.001). However, at low levels of social support (mean-−1 SD), the relationship between learning motivation and cross-cultural adaptability was not statistically significant (B_simple = −0.052, *t* = −1.415, *p* = 0.158).

This pattern implies that social support may act as a necessary condition; in the absence of such support, the positive link between motivation and adaptability is attenuated.

### 4.6 Robustness check

To further verify the reliability of the findings, a series of robustness checks were conducted.

Instrumental Variable Regression (IV Regression): Given the cross-sectional nature of the data, endogeneity (e.g., omitted variable bias) is a potential concern. To address this and better estimate the directional relationship, we utilized Two-Stage Least Squares (2SLS) regression. We selected “whether Arabic was the first choice in the university entrance exam (1 = Yes, 0 = No)” as the instrumental variable.

As shown in Table 7, the first-stage regression results indicated that the instrument significantly predicted learning motivation (β = 0.521, *p* < 0.001, F = 29.63), confirming the relevance of the instrument (F > 10).

**Table 7:** Instrumental variable two-stage least squares (IV-2SLS) regression results.

**Variable**	**First stage**	**Second stage**
Dependent variable	Learning motivation	CCA
Predictor variables	B (SE)	B (SE)
**Instrumental variable**		
First choice	0.521 (0.056)^***^	
**Endogenous variable**		
LM_hat, instrumented		0.630 (0.106)^***^
**Controls**		
Gender	−0.088 (0.066)	−0.016 (0.068)
Grade	−0.026 (0.031)	0.012 (0.027)
**Model diagnostics**		
First-stage *F*	29.63	
Durbin-Wu-Hausman test	12.33^***^	

The dependent variable is cross-cultural adaptability. The values in the table are unstandardized regression coefficients (B).

^***^p < 0.001.

The second-stage results showed that the instrumented learning motivation (LM_hat) maintained a significant positive coefficient on cross-cultural adaptability (B = 0.630, *p* < 0.001). The IV regression coefficient (0.630) was larger than the OLS coefficient (0.319), suggesting that the standard OLS model may have underestimated the magnitude of the relationship potentially due to measurement error or omitted variables. The significant Durbin-Wu-Hausman test (12.33, *p* < 0.001) further supported the use of the IV approach. Although this analysis cannot fully establish causality comparable to experimental designs, the consistency of the IV estimates with the OLS results strengthens the validity of the proposed directional relationship from motivation to adaptability.

Robustness Checks: Subgroup Analysis. To verify the stability of the results across different demographics, we stratified the sample by gender (male vs. female) and grade level, repeating the core moderation analysis for each subgroup.

As shown in Table 8, the main effect of learning motivation (H1) and the interaction effect (H3) remained statistically significant across subgroups. Specifically, the positive predictive relationship of learning motivation on adaptability was significant for both males (β = 0.469, *p* < 0.001) and females (β = 0.357, *p* < 0.001), as well as across grade levels. This indicates that the study's results are robust and not driven by specific demographic sub-populations.

**Table 8:** Subgroup robustness check results.

**Subgroup**	***N***	**Motivation (Beta)**	**Interaction (Beta)**	**Robustness**
**Grade**				
Grade: year 1	110	0.390^***^	0.717^***^	Supported
Grade: year 2	108	0.347^***^	0.620^***^	Supported
Grade: year 3	83	0.438^***^	0.848^***^	Supported
Grade: year 4	14	0.346^*^	0.684^*^	Supported
**Gender**				
Male	74	0.469^***^	0.769^***^	Supported
Female	241	0.357^***^	0.684^***^	Supported

^*^p < 0.05.

^***^p < 0.001.

### 4.7 Further analysis

In Table 9, social support significantly moderated the motivation–adaptability link. The interaction effects were significant for external regulation and intrinsic motivation, indicating that motivation was more strongly associated with cross-cultural adaptability when perceived support was higher. The moderation was more pronounced for external regulation, suggesting that support particularly strengthens the extent to which externally driven motivation translates into adaptive outcomes. Interactions for identified and introjected regulation were not significant in the combined model, implying weaker unique moderation once overlap among motivation types is considered.

**Table 9:** SDT motivation types, social support, and interactions predicting cross-cultural adaptability.

**Variable**	**B(SE)**
**Step 1: controlling variables**	
Gender	0.003 (0.035)
Grade	0.009 (0.017)
**Step 2: independent variables (SDT dimensions)**	
Intrinsic motivation	0.032 (0.052)
Identified regulation	0.136 (0.056)^*^
Introjected regulation	0.095 (0.045)^*^
External regulation	0.126 (0.050)^*^
**Step 3: adjustment variable**	
Social support (SS)	0.460 (0.023)^***^
**Step 4: interactive items**	
Intrinsic motivation × SS	0.213 (0.089)^*^
Identified regulation × SS	0.063 (0.093)
Introjected regulation × SS	0.125 (0.071)
External regulation × SS	0.285 (0.088)^**^
**Model statistics**	
R^2^	0.747
F	81.214^***^

^*^p < 0.05.

^**^p < 0.01.

^***^p < 0.001.

## 5 Discussion

This study examined the relationships between learning motivation, social support, and cross-cultural adaptability among Chinese Arabic language learners. The findings indicated positive associations between both learning motivation and social support with cross-cultural adaptability. Crucially, the results revealed a significant moderating effect of social support, suggesting that the positive link between motivation and adaptability is contingent upon the level of perceived support. Furthermore, further analysis based on Self-Determination Theory (SDT) subtypes indicated that this moderating effect varies across different motivational regulations, providing a nuanced understanding of how internal drive and external resources function synergistically in this specific context.

### 5.1 Theoretical implications

First, the study identifies learning motivation as a significant positive predictor of cross-cultural adaptability (H1). This aligns with the core tenet of Self-Determination Theory, which suggests that behaviors driven by value identification or intrinsic interest may encourage individuals to engage more actively in challenging activities such as cross-cultural interactions (Ryan and Deci, 2017). For Arabic language learners, higher motivation represents not only a desire to learn the language but also a psychological willingness to embrace uncertainty and explore cultural differences (Darvin and Norton, 2021). Notably, our further analysis of SDT subtypes offers a deeper insight. While intrinsic motivation showed a stable association with adaptability, the interaction effect was particularly strong for external regulation. This implies that for students driven by instrumental goals, such as passing exams or job prospects, the presence of social support is especially critical in translating their motivation into adaptive behaviors.

Second, the study suggests that social support serves as an important contextual resource for cross-cultural adaptability (H2). This is consistent with a large body of research on international students' adaptation (Sawir et al., 2008; Rehman et al., 2020). The contribution of this study lies in extending this conclusion to learners in China studying a “distant culture” language. The results suggest that even without being abroad, learning a language with significant cultural differences presents adaptive challenges similar to a “quasi-study abroad” experience, making social support systems crucial for mitigating psychological strain.

The most prominent theoretical contribution is the clarification of the moderating role of social support (H3). Going beyond the additive model of motivation and support, this study reveals an interaction mechanism. The simple slopes analysis demonstrated that at low levels of social support, the predictive power of learning motivation on cross-cultural adaptability was non-significant. This finding provides empirical evidence for the stress-buffering hypothesis in a foreign language context (Cohen and Wills, 1985). It suggests that social support functions as a necessary boundary condition. When support is lacking, even highly motivated learners may struggle to convert their intention into effective adaptation, possibly due to the overwhelming nature of cultural stress. Conversely, high social support appears to facilitate the translation of motivation into adaptive outcomes, supporting an integrated person-environment fit perspective.

### 5.2 Practical implications

Based on the associative relationships and moderating effects observed, this study offers specific pedagogical implications for optimizing Arabic language education.

Targeting High-Risk Groups with Low Social Support The finding that motivation loses its predictive power under low-support conditions suggests a specific risk group: students who are motivated but socially isolated. Educators should not assume that high motivation is sufficient for success. Instead, universities should implement mechanisms to identify these “high motivation, low support” students early. Interventions could include establishing structured peer support groups or “study buddy” systems to ensure that every student is connected to a support network. This would help provide the psychological safety needed for their motivation to be effective and prevent potential adaptation failure.

Differentiated Support for Different Motivational Profiles The further SDT analysis suggests that support strategies should be tailored to students' motivational types. The strong interaction found for external regulation implies that students driven by external pressures, such as career requirements, are heavily dependent on external resources. For these students, teachers should provide instrumental support, such as clear academic guidance and career planning advice, to help them manage pressure. In contrast, for students with high intrinsic motivation, support should focus on emotional validation and creating opportunities for cultural engagement, helping them sustain their interest and translate it into deeper intercultural competence.

Enhancing the Supportive Role of Teachers Given the significant role of social support, the teacher's function extends beyond knowledge transmission to becoming a source of support. Teachers should aim to create a supportive classroom climate that normalizes errors and cultural confusion. By acting as a “significant other” who provides empathetic feedback rather than just corrective feedback, teachers can help buffer the anxiety associated with learning a difficult language like Arabic. This supportive environment can facilitate the positive cycle where motivation leads to successful adaptation practices.

### 5.3 Study limitations and future directions

Although this study provides valuable insights, there are still some limitations that warrant further exploration in future research.

First, the cross-sectional design of this study limits the ability to draw definitive causal conclusions. While the use of Instrumental Variable (IV-2SLS) analysis addressed potential endogeneity and supported the hypothesized directionality, it cannot fully exclude the possibility of bidirectional relationships. For example, successful cross-cultural experiences could reciprocally enhance learning motivation. To gain a deeper understanding of the dynamic relationship between motivation, support, and adaptability, future research should adopt longitudinal designs to track these variables over time, providing more insight into their evolving interactions.

Second, this study relied on self-reported data from students, which introduces the possibility of common method bias and social desirability bias. Although statistical checks, including confirmatory factor analysis (CFA), indicated that these biases were not pervasive in this study, future research should aim for methodological triangulation. This can be achieved by incorporating multi-source data, such as third-party evaluations (e.g., from teachers or peers), objective behavior observations in cross-cultural interactions, or even experimental or quasi-experimental designs to strengthen the validity and causal inference of the findings.

Finally, the use of convenience sampling from a specific cohort of Chinese Arabic learners limits the generalizability of the results to broader populations. While this sampling approach provides high ecological validity for the context of Chinese students learning Arabic, future research should aim to expand the sample diversity. This could include a wider range of university types (e.g., comprehensive universities and specialized foreign language institutions) and geographic regions, allowing for a more comprehensive examination of how school level and regional cultural factors impact the findings. Additionally, future studies can extend the nomological network of cross-cultural adaptability by exploring the moderating role of individual differences, such as personality traits and cognitive styles, and identifying key mediating mechanisms like willingness to communicate.

## 6 Conclusion

In the wave of globalization and the Belt and Road Initiative, cultivating Arabic language talent with excellent cross-cultural adaptability is a key task for China's higher education. This study, through an empirical survey of 315 Chinese university Arabic language learners, clearly reveals the intrinsic mechanism behind the transformation of learning motivation into cross-cultural adaptability and confirms the critical moderating role of social support in this process. The findings indicate that strong learning motivation may be a core factor in driving students' development of cross-cultural adaptability, and a This indicates that there strong social support system could provide 'quality fuel' and 'lubrication' for this engine, potentially making it run more efficiently and sustainably.

The conclusions emphasize that future Arabic language education must move beyond traditional language knowledge teaching and shift toward a more holistic model of education—igniting students' internal motivation and constructing a warm, supportive external environment. Only then can we cultivate future ambassadors who can skillfully navigate between Chinese and Arab civilizations, mastering the language, understanding the culture, and possessing a passionate commitment to long-term success.

## Data Availability

The raw data supporting the conclusions of this article will be made available by the authors, without undue reservation.
